# Compliance with referrals for non-acute child health conditions: evidence from the longitudinal ASENZE study in KwaZulu Natal, South Africa

**DOI:** 10.1186/1472-6963-14-242

**Published:** 2014-06-03

**Authors:** Omolara T Uwemedimo, Stephen M Arpadi, Meera K Chhagan, Shuaib Kauchali, Murray H Craib, Fatimatou Bah, Leslie L Davidson

**Affiliations:** 1 Division of General Pediatrics, Department of Pediatrics, Cohen Children’s Medical Center of New York, New Hyde Park, NY 11040, USA; 2 Department of Pediatrics, College of Physicians and Surgeons, Columbia University, New York, NY, USA; 3 Department of Epidemiology, Mailman School of Public Health, Columbia University, New York, NY, USA; 4 Department of Paediatrics and Child Health, Nelson R Mandela School of Medicine, University of KwaZulu-Natal, Durban, KwaZulu-Natal, South Africa

**Keywords:** Referral, Compliance, Africa, Children, Non acute conditions, Hearing, Vision

## Abstract

**Background:**

Caregiver compliance with referrals for child health services is essential to child health outcomes. Many studies in sub-Saharan Africa have examined compliance patterns for children referred for acute, life-threatening conditions but few for children referred for non-acute conditions. The aims of this analysis were to determine the rate of referral compliance and investigate factors associated with referral compliance in KwaZulu Natal, South Africa.

**Methods:**

From September 2008–2010, a door-to-door household survey was conducted to identify children aged 4–6 years in outer-west eThekwini District, KwaZulu-Natal, South Africa. Of 2,049 identified, informed consent was obtained for 1787 (89%) children who were then invited for baseline assessments. 1581 children received standardized medical and developmental assessments at the study facility (Phase 1). Children with anemia, suspected disorders of vision, hearing, behavior and/or development and positive HIV testing were referred to local health facilities. Caregiver-reported compliance with referrals was assessed 18–24 months later (Phase 2). Relationships between socio-demographic factors and referral compliance were evaluated using chi-square tests.

**Results:**

Of 1581 children, 516 received referrals for ≥1 non-acute conditions. At the time of analysis, 68% (1078 /1581) returned for Phase 2. Analysis was limited to children assessed in Phase 2 who received a referral in Phase 1 (n = 303). Common referral reasons were suspected disorders of hearing/middle ear (22%), visual acuity (12%) and anemia (14%). Additionally, children testing positive for HIV (6.6%) were also referred. Of 303 children referred, only 45% completed referrals. Referral compliance was low for suspected disorders of vision, hearing and development. Referral compliance was significantly lower for children with younger caregivers, those living in households with low educational attainment and for those with unstable caregiving.

**Conclusions:**

Compliance with referrals for children with non-acute conditions is low within this population and appears to be influenced by caregiver age, household education level and stability of caregiving. Lack of treatment for hearing, vision and developmental problems can contribute to long-term cognitive difficulties. Further research is underway by this group to examine caregiver knowledge and attitudes about referral conditions and health system characteristics as potential determinants of referral compliance.

## Background

The improvements in overall childhood mortality in low and middle-income countries have been accompanied by a growing burden of chronic conditions among older, surviving children [1,2]. The management of chronic conditions during childhood relies heavily on well-functioning referral systems that support effective transitions from screening to treatment [3,4]. Effective referral systems are instrumental in improving quality of care, decreasing healthcare expenditures and improving health outcomes [5].

Referral compliance is an important indicator of an effective referral system [4] reflecting a cascade that involves multiple steps from initial recognition of a problem through access to, and utilization of, specific health services. Although high referral compliance is essential for optimizing the health of children with non-acute conditions, very few published studies have examined referral compliance in sub-Saharan Africa. Previous studies have focused on children under 5 years of age and address referral compliance for acute rather than non-acute or chronic conditions [6-10]. Studies evaluating referral compliance for non-acute and chronic conditions, such as neuro-developmental disability and HIV, for school-aged children living in sub-Saharan Africa are sparse.

Strengthening referral compliance is a priority in disadvantaged communities in South Africa, a post-apartheid country plagued by endemic poverty, low educational attainment, overburdened health facilities, human resource shortages and geographic isolation from health services [11]. Beginning in the mid-1990s, South Africa implemented the District Health System (DHS), following the guidance of the World Health Organization, to achieve optimal decentralization of health services. Under this system, health services are provided through a stepwise approach, starting with a network of local health clinics and centers at the primary level with referral to higher-level facilities, as needed. However, the current health system has not guaranteed efficient completion of referrals for health problems [11].

Understanding factors that contribute to compliance with referrals for healthcare is a critical step in developing interventions to improve these referral systems. According to the Aday and Andersen model for access and utilization of care, multiple factors may influence utilization of health services. Such factors may include characteristics of the population at risk and characteristics of the health delivery system [12].

In this study, we examine rates of compliance with referrals to local health facilities for pre-school children who were participants in an epidemiologic study conducted in peri-urban KwaZulu Natal, South Africa. Our aims were to: 1) describe the pattern of referral for non-acute conditions; 2) to describe the overall rate of referral compliance; and 3) to identify factors associated with referral compliance for children living in this disadvantaged area of KwaZulu Natal, South Africa.

## Methods

Compliance with referral for non-acute health problems was assessed in a sample of children enrolled in the ASENZE study. The ASENZE study is a 2-phase longitudinal, population-based cohort study designed to determine the prevalence of neuro-developmental disability and identify potential health, contextual and psychosocial predictors of disability among children living in a semi-urban area in KwaZulu Natal, South Africa. This population was chosen since potential risk factors (e.g., poverty, low parental educational attainment, high unemployment rates) for developmental disability are highly prevalent.

In Phase 1, completed between 2008 and 2010, a door-to-door survey of all households in five contiguous isiZulu tribal areas was conducted and all 4–6 year old children (n = 2,049) were invited to a comprehensive medical and developmental assessment. Age of the child was determined by caregiver recall of birthdate and when available, by the child’s immunization card. After obtaining informed consent from an adult primary caregiver/biological parent, 1,787 (87%) children were enrolled. Household socio-demographic data were obtained in the field and all enrolled children and caregivers were invited to a full assessment within 2–3 weeks at the study assessment center. 1,581 (89%) of the 1,787 enrolled children received comprehensive medical and developmental assessments. The child’s comprehensive medical and developmental assessment took place at the AZENZE study research facility and included a semi-structured pre-coded medical history and physical exam with a developmental component adapted for use for this study [13]. In addition, all children underwent a hearing screen using Distortion Product Otoacoustic Emissions (DPOAE). Those who failed the DPOAE also had tympanography by emittance audiometry. Vision screening was performed using a “tumbling E” Snellen eye chart. HIV counseling and voluntary testing were offered to all participants and performed after informed consent was signed by the caregiver. Hemoglobin was measured by fingerstick testing. Children with any of the following were referred: anemia (Hemoglobin <10 g/dL), vision impairment (Snellen test below 20/20), hearing (inability to detect frequencies between 2–5 kilohertz on DPOAE) or middle ear problem (abnormal pressure-compliance curve on tympanometry), and other problems, detected on the medical and developmental assessment.

During the Phase 1 assessment, 516 children were identified as having ≥1 referral condition. The caregivers of these children were educated about all referral conditions and given referrals to the health facility closest to their home for further management. Referral instructions were given verbally and in writing, and caregivers were also given a referral letter explaining the reason for referral. Caregivers were instructed to bring this letter when the child was taken to the referral facility. The single most important referral (i.e., the primary referral), was determined by the study physician and reported to the caregiver. Referrals were made for any additional evaluation or treatment of the child’s health problem because this could not be performed within the research setting.

**Figure 1:**
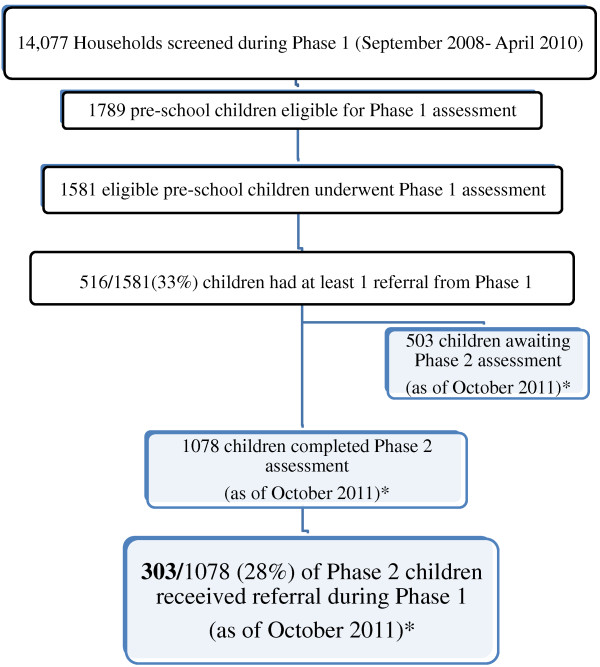
**Children included in final analytic sample.** *Phase 2 assessment still ongoing at the time of analysis.

The referral network included 7 primary health clinics and community health centers within a 5 km radius of Kwadadengendlale and 4 area referral hospitals. Clinical services and referral procedures had been confirmed for each of the referral facilities prior to the initiation of the study.Children were scheduled for a second assessment (Phase 2) 18–24 months following phase 1 assessment, as of September 2012. At the time of this analysis, 1078 of the 1581 children assessed in phase 1 had completed a follow-up assessment. Among those who completed their Phase 2 assessment, 303 were children who had received a referral during Phase 1. Therefore, this analysis is based on only 303 of the 516 children who received a referral in Phase 1 (Figure 1).

Confirmation of completion of the referral (yes/no) and assessment of factors that facilitated or prevented completion of the referral were ascertained by means of a semi-structured questionnaire conducted by a trained research assistant.

The ASENZE study was approved by the Biomedical Research Ethics Committee of University of KwaZulu-Natal, Durban, South Africa, the Institutional Review Board of Columbia University, New York, USA.

### Study setting and population

The study was conducted in Kwadedangendlale, a peri-urban area comprised of isiZulu tribal areas located 40 kilometers northwest of Durban in the KwaZulu-Natal province [14]. In 2008, over half of the adult population was unemployed and one-fifth of caregivers for children had no formal education [14]. HIV infection prevalence rates are high throughout the province, with rates as high as 40% reported among antenatal clinic attendees [15].

The eThekwini Health District is responsible for the public health services in this area. The health care services throughout the entire Health District are provided through a multi-tiered system, which is comprised of 115 primary health care clinics, 8 community health centers, 2 state-supported hospitals and 16 district and provincial hospitals that are tertiary or quaternary-care facilities [16]. In Kwadadengendlale, healthcare services are limited to 7 clinics, 2 community health centers, and 1 district hospital. Informal health services provided by traditional healers are also used in the study area [17].Of the 1581 children who were assessed during Phase 1, 516 (32.6%) received at least one referral for further evaluation or treatment of non-acute conditions. As of October 2011, 1078 children had returned for Phase 2, including 303 who had one or more referrals for evaluation or treatment of a non-acute condition during Phase 1. The flow of study subjects included in this analysis is presented in Figure 1. Referrals for mild anemia, suspected visual acuity disorders and dental problems were referred to primary health care clinics or community health centers (i.e., first-level health facilities). Behavioral problems, developmental delay, disorders requiring speech, physical or occupational therapy were referred to the district or regional hospital. In addition, children found to have a positive HIV test were referred for further evaluation at the district hospital (which included HIV staging and CD4 count testing in order to assess the child’s need for antiretroviral therapy).

### Measures

Referrals were categorized as follows: mild anemia, visual acuity disorders, hearing or middle ear disorders, behavioral disorders, developmental delay, problems requiring speech, physical or occupational therapy, HIV infection, dental problems and other disorders as determined by the study physicians.

The compliance with the primary referral made for the children during Phase 1 as reported by the caregiver was ascertained during Phase 2. Referral compliance was defined as the proportion of children reported during the Phase 2 assessment to have attended the referral facility recommended for the primary problem during Phase 1. In order to measure poverty level, a household asset index was created. A relative index of household assets was computed using information on the households for all 1581 children who had a Phase 1 assessment. This followed the approach of the DHS surveys using combined household-level information on land ownership, possession of durable goods and household dwelling characteristics (building material, sources of drinking water, type of toilet facility and main source of cooking fuel,). We used principal components analysis to define the weights, where the index is the first principal component, since it summarizes the largest amount of information (16% of the variance). The index was then stratified into three categories top 20%, middle 40% and lowest 40%. This approach has been used in the Demographic and Health Surveys and has been previously described in the literature [18].

### Data analysis

Frequencies were used to report referral compliance and chi-square tests were performed to examine the relationship between socio-demographic factors including patient demographics (e.g., child age at time of referral, caregiver age, child gender) and household demographics (e.g. wealth status) [9]. Associations between referral characteristics and referral compliance were also assessed.

All analyses were performed using SPSS version 19.0 (SPSS Inc. Chicago, Ill).

## Results

The demographic characteristics of children given referral (n = 303) are presented on Table 1. The mean age (SD) at the time of referral was 5.0 (0.6) years and slightly more than half were female. The mean age of the caregivers was 39 (14.1) years and one-third of caregivers was someone other than the child’s birth mother. The majority (74%) of caregivers had attained a secondary school education or lower.

**Table 1:** Characteristics of study sample referred to an outside health facility (N = 303)

**Child and caregiver characteristics**	**Mean age in yrs (SD, range)**
Child age	5.0 (0.6, 3.7-6.0)
Caregiver age	39.7 (14.1, 20.0-81.0)
	**N (% of sample)**^**a**^
Child gender	
*Male*	147 (48.5)
*Female*	156 (51.5)
Type of primary caregiver^a^	
*Other family member*	99 (32.7)
*Birth mother*	197 (65.0)
Household wealth status^b^	
*Richest 20%*	65 (21.5)
*Middle 40%*	85 (28.0)
*Poorest 40%*	153 (50.5)
Highest educational attainment of any household member^a^	
*≤Secondary School*	225 (74.3)
*>Secondary School*	30 (9.9)
Same caregiver in both Phase 1 and Phase 2 assessments^a^	
*Yes*	243 (82.5)
*No*	46 (14.9)
**Referral characteristics**	**N (% of sample)**^**a**^
**Primary referral problem**^**a**^	
Mild anemia	41 (13.5)
Hearing or ear problem	68 (22.4)
Vision problem	36 (11.9)
Problem requiring occupational, speech or physical therapy	2 (0.7)
Behavioral problems	7 (2.3)
HIV	20 (6.6)
Developmental delay	14 (4.6)
Dental problem	11 (3.6)
Other	103 (34.0)
**Type of facility referred to (level of care)**^**a**^	
Local health clinic (primary)	128 (42.2)
Community health center (primary)	4 (1.3)
District hospital (secondary)	3 (1.0)
Regional hospital (tertiary)	96 (31.7)
Other	6 (2.0)
Unknown	64 (21.1)

^a^% of sample may not equal 100% due to missing data.

^b^% of overall study sample (all 1581 children who completed phase 1 assessment).

The most common primary referrals were mild anemia (14%), hearing or middle ear disorders (22%) and visual acuity disorders (12%). Chronic asthma, eczema and fungal skin/scalp infections accounted for 20% of referrals. Almost half of children were referred to their local health clinic, while a third were referred to the regional hospital (Table 1).

Compliance with the primary referral was reported by less than half (45%) of caregivers. Caregivers were significantly more likely to comply with referrals for HIV infection (60%) and anemia (61%) and least likely for hearing/middle ear disorders, visual acuity disorders and developmental delay referrals (Table 2). Children who lived in a household where any household member had greater than a secondary school education were also significantly more likely to have complied with referrals than children in households where educational attainment was lower (77% vs. 40%; p < 0.001). In addition, younger caregivers were also more likely to have higher compliance than older caregivers (49% vs. 36%, p < 0.05). Although household wealth status was not significantly associated with referral compliance, the trend was significant where those in the middle and poorest tertiles were least likely to comply with referrals (p = 0.044). The number of children in the household was not associated with referral compliance. Additionally, children whose primary caregiver did not change between the first and second study phase assessments were more likely to have a completed referral (50% vs. 20%; p < 0.001).

**Table 2:** Association between referral compliance with referral characteristics and socio-demographic factors (N = 303)

**Covariate**	**Completed/total referrals (N)**^**a**^	**Referral compliance (%)**	**Crude OR**	**95% CI**
**Overall**	135/303	44.6		
**Demographics**				
Child age at time of referral				0.83-2.12
*≥5 years*	67/160	41.9	Ref	
*<5 years*	62/127	48.8	1.32	
Child gender				0.94-2.33
*Female*	62/155	40.0	Ref	
*Male*	73/147	49.7	1.48	
Caregiver age				1.05-2.77
*≥40 years*	41/115	35.7	Ref	
*<40 years*	84/173	48.6	1.70*	
Type of primary caregiver				0.63-1.66
*Birth mother*	88/196	44.9	Ref	
*Other family member*	45/99	45.5	1.02	
Household wealth status^c^				0.29-1.07
*Richest 20%*	37/65	56.9	Ref	0.29-0.93
*Middle 40%*	36/85	42.4	0.56	
*Poorest 40%*	62/152	40.8	0.52*	
Highest educational attainment of any member				2.02-11.88
*≤Secondary School*	90/224	40.2	Ref	
*>Secondary School*	23/30	76.7	4.89***	
Same caregiver in both Phase 1 and Phase 2 assessments				1.85-8.65
*No*	9/45	20.0	Ref	
*Yes*	125/250	50.0	4.00***	
**Primary referral problem**			N/A^b^	N/A^b^
Mild anemia	25/41	61.0		
Hearing or ear problem	35/68	51.5		
Vision problem	18/36	50.0		
Problem requiring occupational, speech or physical therapy	1/2	50.0		
Behavioral problems	0/7	0.0		
HIV	12/20	60.0		
Developmental delay	7/14	50.0		
Dental problem	4/11	36.4		
Other	32/102	31.4		
**Referral facility type (level of care provided)**			N/A^b^	N/A^b^
Local health clinic (primary)	70/128	54.7		
Community health center (primary)	2/4	50.0		
District hospital (secondary)	2/3	66.7		
Regional hospital (tertiary)	54/96	56.3		
Other	3/6	50.0		

^a^For each bivariate association, denominators may vary because of missing data.

^b^Not applicable for chi-square tests due to low expected counts in at least one category.

*p < 0.05; **p < 0.01; ***p < 0.001.

^c^% of overall study sample (all 1581 children who completed phase 1 assessment).

## Discussion

In this study, overall compliance rate for children with suspected non-acute conditions was 45%. Although there are few studies evaluating referral compliance for children in sub-Saharan African settings with which to compare, the compliance rate observed is clearly inadequate for optimal child health and development outcomes in this population. One possible contribution to the low rate of referral compliance may be that some referrals were not necessarily prompted by caregiver complaints, but were identified during the comprehensive medical and developmental examination. This may have reduced compliance rates, since the caregiver may have been less motivated to seek further evaluation due to the absence of acute, physical changes in the child.

A substantial proportion of children in this community warranted referral for one or more non-acute conditions that, without proper interventions, could impair long term well-being including mild anemia, poor visual acuity or hearing disorders. Anemia, including common forms secondary to dietary insufficiencies, negatively impact exercise tolerance, physical growth, as well as neurodevelopment and cognitive function [19]. Reduced visual acuity significantly affects participation and learning in classroom settings and can contribute to poor academic achievement. Furthermore, certain causes of visual impairment during childhood are a cause of later life blindness [20]. In addition, prior studies indicate that a considerable burden of poor school achievement among African children is attributable to hearing or middle ear problems [21,22].

The referral compliance observed in this study varied by the type of problem with higher compliance for HIV infection and anemia while those for neuro-developmental problems (vision, hearing, developmental delay) were consistently low. Although we were unable to evaluate possible explanations for this, previous research indicates that referral compliance is greater for children referred for conditions with acute, life-threatening symptoms [6]. Therefore, the higher compliance with children referred for HIV infection may be due to this condition being perceived as a potentially life-threatening condition. The prevalence of HIV in this region of South Africa is among the highest in the country and antiretroviral therapy is increasingly accessible. Forty percent of pregnant women attending antenatal clinics in KwaZulu-Natal are HIV-infected. Thus, caregivers in this community likely appreciate the importance of obtaining medical treatment for HIV-infected children [15]. A lack of trust in screening results, lack of knowledge of the illness, and fear of diagnosis or treatment are reported to be most common reasons for unsuccessful referral for children with vision problems [23]. Additional analyses conducted with this study population are underway to examine the influence of caregiver knowledge and attitudes on compliance with referral.

Furthermore, health system factors, such as limited availability of treatment services, are likely to be contributing factors to low referral compliance. Although health systems factors were not evaluated, during the study, the main referral facility for children with visual acuity problems experienced intermittent closures. This may have been an important deterrent to compliance with referrals for visual acuity disorders.

Referral compliance was also noted to be higher for children with younger caregivers in comparison to older caregivers in our study. The majority of caregivers <40 years were noted to be the child’s biological mother, while the majority of older caregivers were identified as biological grandparents. Possible reasons for the relationship between poorer compliance and older caregiver age includes the fact that grandparents acting as primary caregivers of children tend to take on this role because of a lack of younger adult family members available to fulfill this role. These elderly caregivers usually have far fewer social supports than their younger counterparts to assist them with resources to facilitate accessing health services for children when needed [24]. In addition, although economically active, most elderly caregivers have higher rates of illness and disabling condition than their younger counterparts, which may prevent them from bringing children to care [24,25].

Similar to other studies conducted in Africa, referral compliance was found to be strongly associated with household educational attainment [26].The effect of caregiver education on utilization of health services for children in African populations has been previously reported [27-29]. The theoretical linkage between education and compliance may be a result of heightened understanding of the referral problem and awareness of the need for further care. Additionally, education may be a proxy for increased access to resources, allowing the caregiver to more easily overcome potential barriers to referral compliance (e.g. cost, transport). In the present study however, we were unable to assess whether the relationship between education and compliance remained significant, even after adjusting for household assets. Further analyses would be needed to determine if the association between referral compliance and household education is due to heightened awareness of the importance of intervention versus increased access to essential resources for completing referrals.

This study also found that referral compliance was lower among children whose primary caregiver changed over the two years between Phase 1 and Phase 2. We were unable to distinguish whether poor compliance among children with changing caregivers is simply due to lack of knowledge about prior referral compliance by the new caregiver or true non-compliance with the referral. However, in South Africa, lack of continuity in caregiving is highly prevalent, due in part to the high rate of parental death secondary to HIV infection as well as economic factors [30]. In this sample, nearly one-third of primary caregivers were someone other than the birth parent. Therefore, in communities with high HIV prevalence or other socially disruptive conditions, establishing the extent to which change in the primary caregiver for a child disrupts receipt of health services for children appears to be important for further study and a potential target for intervention.

There are a number of limitations in this study. Referrals were initiated within a research study by a research physician apart from the health system and thus, the observed patterns may not be a true reflection of patterns of referrals or referral compliance seen in the public health services sector. We did not assess the referral facility type (i.e. primary vs. tertiary) and reputation, which may have influenced compliance referral. We also relied on caregiver report to assess compliance, which is subject to recall bias, and did not have access to health facility records to validate these findings independently. Also, without regression analyses, we were unable to control for potential confounders of the significant relationships identified through chi-square tests, such as caregiver knowledge, attitudes and beliefs about the specific referral or referral site. In addition, clustering effects may have introduced bias into our sample since referral compliance patterns among households with more children may have been overrepresented. Our study population resides in a semi-rural setting, where differences in health facility density and road/public-transportation networks may limit the generalizability of these findings [31]. Lastly, it is important to note that referrals made within our study setting for easily treatable conditions (e.g. anemia, skin conditions) would not require referral at the primary health care level since treatment is readily available in these settings.

## Conclusion

Our findings demonstrate that compliance with referrals is sub-optimal for neuro-developmental problems, including hearing and vision problems. Children who have younger caregivers, caregivers with lower educational attainment and those with changing caregivers are at increased risk for poor referral compliance. Additional studies are in progress by this study group using regression analyses to identify other possible contributory factors including: caregiver knowledge and attitudes about referrals, environmental factors (e.g. financial and geographical accessibility) and health system factors (e.g. service availability, health worker availability and health system responsiveness). Additional research in this area is essential to developing interventions to strengthen referral processes as a means to improve the *quality* of life for disadvantaged African children.

## Competing interests

The authors declare that they have no competing interests.

## Authors’ contributions

OTU, SMA, MKC, SK, MHC, LLD participated in the design of the analysis. OTU performed statistical analysis and drafted the first manuscript. OTU, MKC, SK and MHC were involved in data collection. FB participated in data analysis and the drafting of the manuscript. SMA, MKC, SK, MHC and LLD assisted in interpretation and drafting of the manuscript. All authors read, commented on and approved the final manuscript.

## Pre-publication history

The pre-publication history for this paper can be accessed here:

http://www.biomedcentral.com/1472-6963/14/242/prepub
